# The Response of the Human Umbilical Vein Endothelial Cell Transcriptome to Variation in Magnesium Concentration

**DOI:** 10.3390/nu14173586

**Published:** 2022-08-31

**Authors:** Lujain A. Almousa, Andrew M. Salter, Marcos Castellanos, Sean T. May, Simon C. Langley-Evans

**Affiliations:** 1 Department of Health Sciences, College of Health and Rehabilitation Sciences, Princess Nourah Bint Abdulrahman University, P.O. Box 84428, Riyadh 11671, Saudi Arabia; 2 Division of Nutritional Sciences, School of Biosciences, The University of Nottingham, Nottingham NG7 2TU, UK; 3 Nottingham Arabidopsis Stock Centre, School of Biosciences, The University of Nottingham, Nottingham NG7 2TU, UK

**Keywords:** magnesium, vascular endothelium, microarray, transcriptome

## Abstract

Vascular endothelial cells have a critical role in the maintenance of cardiovascular function. Evidence suggests that endothelial function may be compromised under conditions of magnesium deficiency, which increases vulnerability to inflammation. Whole genome transcription analysis was used to explore the acute (24 h) effects of magnesium on human umbilical vascular endothelial cells (HUVEC) cultured in low (0.1 mM) or high (5 mM) concentrations. With low magnesium 2728 transcripts were differentially expressed compared to the 1 mM control cultures and 3030 were differentially expressed with high magnesium. 615 transcripts were differentially expressed under both conditions, of which only 34 showed a concentration-dependent response. Analysis indicated that cellular organisation and biogenesis and key cellular processes such as apoptosis were impacted by both low and high conditions. High magnesium also influenced protein binding functions, intracellular signal transduction, metabolic and catalytic processes. Both conditions impacted on stress-related processes, in particular the inflammatory response. Key mediators of calcium-dependent regulation of gene expression were responsive to both high and low magnesium conditions. The HUVEC transcriptome is highly sensitive to acute changes in the concentration of magnesium in culture medium. The findings of this study support the view that whilst inflammation is an important process that is responsive to magnesium, the function of the endothelium may be impacted by other magnesium-induced changes including maintenance of cellular integrity, receptor expression and metabolic functions. The high proportion of transcripts that did not show a concentration-dependent response suggests variation in magnesium may elicit indirect changes, possibly mediated by other ions.

## 1. Introduction

Vascular endothelial cells play a key role in controlling vascular function through regulation of blood flow [1], cellular adhesion, vascular inflammation, vessel tone and smooth muscle proliferation [2]. Key early steps in atherosclerosis and thrombosis involve injury or dysfunction of the endothelium. Vascular endothelial cell inflammation is the most important driver of atherosclerosis [3]. Cultured endothelial cells have been used extensively as an in vitro model in pathological and physiological experiments relating to vascular disease [4]. Gene expression in cultured endothelial cells has been shown to be responsive to a variety of nutritional stressors and challenges, including micronutrients and plant derived polyphenols [5,6,7]. In human populations the range of Mg intakes from food and in water is broad. Whilst hypermagnesemia is relatively uncommon, disorders related to poor magnesium status are a common problem worldwide [8]. The National Diet and Nutrition Survey in the United Kingdom reported that approximately 10% of men and 9% of women, aged 19 to 64, consumed less than the lower reference nutrient intake (LRNI), which is often an indicator of widespread deficiency in the population [9]. There are other causes of magnesium deficiency, such as malabsorption, gastrointestinal and renal loss, liver or pancreatic disease, vomiting, diarrhoea and chronic alcoholism. Moreover, hypomagnesaemia may be induced by diabetes due to excess excretion associated with diuresis [10]. Magnesium (Mg) is one micronutrient of potential interest in terms of vascular endothelial cell function, as epidemiological and experimental animal studies suggest that poor intakes of magnesium are associated with a greater risk of coronary heart disease [11]. In vitro, endothelial growth is impacted by magnesium deficiency through an increase in cytokine release in conditions of low magnesium concentration [12]. Magnesium status may impact on cardiovascular health through a number of mechanisms [13]. Our previous work suggests that low extracellular magnesium concentrations have a negative influence on endothelial cell proliferation, increase monocyte adhesion [14], inhibit cell migration [15], and exacerbate the response to inflammatory challenges [16]. These effects appear to be due to the activation of many types of cytokines, which induce overexpression of the inflammatory phenotype in endothelial cells [17] and are observable even after 24 h of exposure to variation in magnesium concentration. The cytokines orchestrate the inflammatory response to infection and injury, and play an important role in vascular endothelial cell biology. IL-8 is a critical cytokine that attaches to neutrophils and moves them to the inflamed area. IL-8 expression depends on the activation of NF-κB [18,19,20], and inflamed endothelial cells are the main producers of IL-8 [21]. In addition, GRO and GROα work with IL-8 in attracting neutrophils [22]. MCP-1 cytokines also bind to mononuclear leukocytes [23]. Moreover, IL-6 directs the generation of thrombocytes and the differentiation of B cells [24].

Such mechanisms have been identified through a candidate-led approach which may miss other changes that are associated with low or high magnesium concentrations. To date, there has been no systematic approach to understanding the impact of magnesium on the vascular endothelial cell transcriptome, an important first step towards fully determining the mechanisms which underpin the functional effects of magnesium. Therefore, this study aimed to perform an unbiased analysis of the acute effect of varying magnesium concentrations on the full transcriptome of human umbilical vascular endothelial cells (HUVECs).

## 2. Materials and Methods

### 2.1. Cell Culture 

Primary HUVECs (C2519A; Lonza Basel, Basel, Switzerland) were cultured in endothelial cell growth medium (EGM-2, Lonza) with 2% fetal bovine serum (FBS), following the manufacturers instructions. The HUVEC cultures were incubated in six-well plates at seed density (7500/cm^2^), at 37 °C (5% CO_2_), with the medium changed every other day until the cells were grown to 80–90% confluence. At this point, the HUVECs were cultured for 24 hrs in a human endothelial Mg-free medium (Invitrogen, Waltham, MA, USA) supplemented with 10% FBS, 1% penicillin 100× 1%, 5% endothelial cell growth supplement (Sigma-Aldrich, Poole, UK), and MgSO_4_ concentrations of 0.1 mM or 5 mM following the method of Ferre et al., and Maier et al., [25,26]. The optimal concentration of FBS to use with the experimental medium, which lacked the growth factors present in the basal EGM-2, and the incubation period required to alter the cellular response to challenges, was determined through preliminary experiments. The samples were compared with cells cultured with 1 mM MgSO_4_, which is the physiological circulating concentration of Mg^2+^.

### 2.2. RNA Extraction 

RNA was extracted from the HUVECs using a High Pure RNA Isolation Kit (Roche, Mannheim, Germany) according to the manufacturer’s protocol. A Thermo Scientific NanoDrop ND-1000 spectrophotometer (Thermo Scientific, Wilmington, NC, USA) was used to quantify and determine RNA concentrations. RNA integrity was estimated using an Agilent RNA 6000 Nano Kit, 2100 Bioanalyzer, and Agilent 2100 Expert Software following the manufacturer’s instructions (Agilent Technologies, Waldbronn, Germany). The RNA integrity number (RIN) was measured using Agilent 2100 Bioanalyzer. All RNA preps showed RIN ≥ 9.2, where >7 is the accepted value.

### 2.3. Microarray Analysis 

Whole-genome transcriptome analysis was conducted by hybridizing three biological samples of total RNA per treatment to the GeneChip^®^ Human Genome U133 Plus 2.0 Array (#900470, Affymetrix, High Wycombe, Bucks, UK). All steps of sense cDNA synthesis, fragmentation, and hybridization were performed according to the manufacturer’s protocol (GeneChip^®^ 3000 System, Affymetrix, Cleveland, OH, USA). 

### 2.4. Bioinformatics and Statistical Analysis

Gene expression profile data was generated as CEL files and initially subjected to analysis by the Partek Genomics Suite 6.6 software. Quality Control (QC) metrics were checked by examining surface defects, hybridization, labeling, and a ratio of the 3′ probe set to the 5′ probe set (3′/5′ ratio) to provide the quality of the microarray data. The values were log^2^ transformed and quantile normalization using the Robust Multi-array Average (RMA). The list of genes of interest comprised genes up-regulated or down-regulated by at least one-fold (log2 fold-change) with an unadjusted *p*-value < 0.05. To analyse the effects of magnesium treatment, the high-Mg^2+^ and low Mg^2+^ groups were compared to the physiological concentration, 1 mM. Data in this paper are reported as Log2 fold-change. Transcript expression data was used to identify biological and molecular functions, which showed significant enrichment by accessing the Gene Ontology (GO) Consortium bioinformatics resource using appropriate portals (Ingenuity Pathway Analysis, Partek Genomics Suite, and PANTHER) [27]. 

## 3. Results

### 3.1. Microarray Approach: Effect of Magnesium Concentration on the HUVEC Transcriptome 

**Figure 1:**
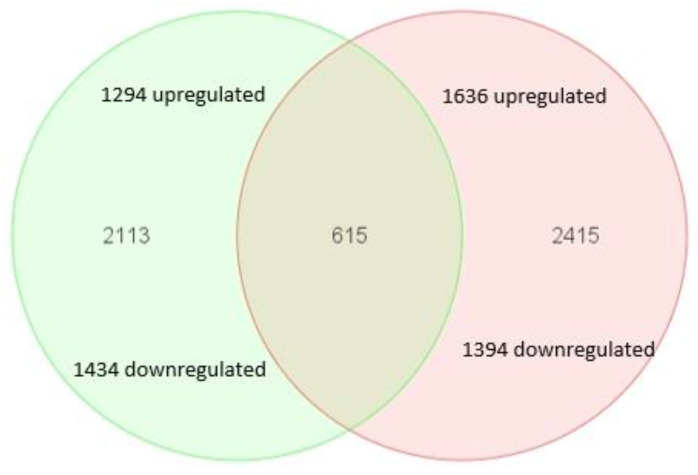
Venn diagram of HUVECs genes that were significantly expressed after culturing for 24 h in 0.1 mM MgSO_4_, indicated by green, and 5 mM MgSO_4_, indicated by red, as compared to 1 mM MgSO_4_. 615 genes were differentially expressed relative to control in both conditions.

The microarray data were initially examined using principal component analysis, which showed a clear separation between mRNA expression across the three groups (data not shown). Low variance was observed, indicating well-conserved mRNA expression within each group. 5758 out of approximately 47,000 transcripts present on the U133 Plus 2.0 Array chip were differentially expressed in the high- and low-magnesium treatments as compared to the control. Figure 1 shows that 2728 transcripts were differentially expressed after the HUVECs were cultured in 0.1 mM MgSO_4,_ of which 1434 were down-regulated, and 1294 were up-regulated. HUVECs cultured in 5 mM MgSO_4_ expressed 3030 transcripts differently to the control, with 1394 being down-regulated and 1636 up-regulated (Supplementary Table S1). Six hundred and fifteen transcripts were differentially expressed in both the high- and low-magnesium treatments relative to the control (307 up-regulated, 308 down-regulated; (Supplementary Table S2). Table 1 and Table 2 list the top 20 genes that were differentially expressed in HUVECs cultured in 0.1 and 5 mM MgSO_4_ based on Log2 fold change. 

**Figure 2:**
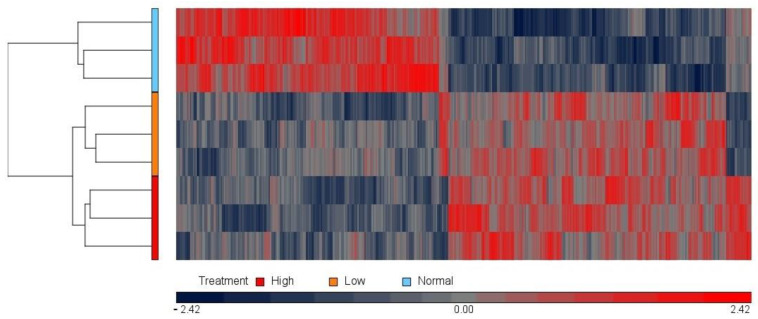
Hierarchical clustering of the 615 genes that were commonly expressed in high- and low-magnesium treatments, with blue blocks representing down-regulated expression and red blocks representing upregulated expression. Each row represents one sample, and each column represents one gene. The red bar on the side of the heat map represents the 5 mM MgSO_4_ group, *n* = 3. The blue bar represents the 1 mM MgSO_4_ group (control), *n* = 3. The orange bar represents the 0.1 mM MgSO_4_ group. The scale at the bottom of the diagram represents Log2 fold-change values.

Before examining in detail which genes, processes, and pathways were differentially expressed in low- or high-magnesium conditions, a sub-analysis was conducted to assess the 615 transcripts that were differentially expressed in both conditions, Figure 2 shows the hierarchical cluster analysis (heat map) of these 615 transcripts, which revealed distinctly different expression patterns between the control (1 mM MgSO_4_) and the high- and low-magnesium treatment groups. As is clear from the heat map, most of these transcripts (581 transcripts) were up-or down-regulated in both low- and high-magnesium conditions, rather than the expected opposing effects of the two treatments. The Gene Ontology (GO) Consortium bioinformatics resource (GOC, http://www.geneontology.org/ 1 July 2022) was used to classify and describe functions of the transcripts that were differentially expressed in both high- and low-magnesium conditions. This analysis showed that the two conditions had effects consistent with changes to cell component organisation (*p* = 2.49 × 10^−5^), anion binding (*p* = 1.15 × 10^−4^), and protein binding (*p* = 7.9 × 10^−10^), and nucleic acid binding (*p* = 8.7 × 10^−3^). 

### 3.2. Gene Ontology Analysis

Table 3 and Table 4 show the enrichment scores and numbers of genes associated with significantly enriched GO processes for the full datasets. All transcript expression data is provided in Supplementary Table S1 which shows all of the differentially expressed transcripts, with the fold-change and is searchable. In terms of biological processes, low magnesium conditions markedly impacted RNA splicing (590 transcripts), cellular processes (819 transcripts), and cellular component organisation and biogenesis (243 transcripts), whilst high magnesium led to significant enrichment of cellular processes (1052 transcripts), metabolic processes (748 transcripts), cellular component organisation and biogenesis (296 transcripts), primary metabolic processes (604 transcripts), cell adhesion (68 transcripts), phosphate-containing compound metabolic processes (228 transcripts), intracellular signal transduction (160 transcripts), cell death (65 transcripts), apoptosis (62 transcripts), G coupled receptor signalling (24 transcripts), nucleobase-containing compound metabolic processes (364 transcripts) and perception of chemical signals (6 transcripts). 

No molecular functions were enriched in conditions in low magnesium at an FDR of *p* < 0.005 (Table 4). With high magnesium, significant enrichment was noted for catalytic activity, binding activity, protein binding, and hydrolase activity (Table 3). Binding was heavily related to metal iron binding (522 transcripts) and ATP binding (226 transcripts). Conditions of high magnesium significantly influenced the expression of 547 transcripts associated with catalytic activity. The majority of these were hydrolases (251 transcripts; e.g., Ggh, Abdh, Atic, Abhd1, Hibch, Sgsh, Ddah2), and the range of enzymes responding to magnesium mainly included those involved in protein and peptide metabolism (e.g., Sppl3, Sppl13, Usp34, Usp36, Tpp1), and carboxylic acid metabolism (e.g., Csad, Mccc2, Paics). Cofactor binding was also influenced by high magnesium (e.g., Hpgd, Scp2, Gale, Mutyh1, Csad, Lancl1, Acly, Phyh), although there was no evidence that these effects were limited to enzymes with magnesium at their catalytic centers. All parts of the cell (intracellular, cytoplasm, nucleus, organelle, ribosome-associated GO processes) were impacted by the variation in magnesium concentrations).

The GO process Cellular Processes was significantly enriched under both low and high magnesium conditions (Table 3 and Table 4). This term covers a broad range of processes, including cell growth and maintenance, cell cycle, cell death, cellular communication, signal transduction, and immune system processes, many of which were separately identified as significantly enriched with high magnesium. As magnesium concentration is known to impact on the proliferation and viability of HUVECs in culture analysis principally focused on cell death and apoptosis. Of 65 transcripts identified as differentially expressed with high magnesium, 19 were also differentially expressed with low magnesium. Interestingly 8 were up-regulated in both conditions and were 7 down-regulated. Four transcripts were of particular interest as they showed different expression changes with low and high magnesium (Pcbp2, Pak4, MAP2K6 up-regulated in high magnesium, down-regulated in low; Cflar down-regulated with high magnesium, and up-regulated with low). MAP2K6, Pak4, and Pcbp2 are all associated with cell proliferation, whilst Cflar is an inhibitor of apoptosis.

Of 160 transcripts associated with intracellular signal transduction differentially regulated in high magnesium conditions, 31 were also differentially regulated with low magnesium (17 up-regulated in both states and 8 down-regulated in both states). Two (Ddr2 and Cflar) were down-regulated with high magnesium and down-regulated with low magnesium, whilst 4 were up-regulated with high and down-regulated with low (Smad2, Arhgap18, Pak4, and MAP2K6). Among transcripts that were differentially regulated in both conditions were NFATC1 (up-regulated) and NFATC3 (down-regulated) which encode transcription factors that play a critical role in responses to calcium. Several of the intracellular signaling transcripts were found to be calcium-sensitive or functionally related to calcium (e.g., Myo1c, Myh7B, Dab2, Grm1, Smad3, Ccl4) and a key steps in the calcium-sensing and gene regulation pathways were also differentially regulated by magnesium (Ryr1 down-regulated in both states; Ryr2 down-regulated with low magnesium; calmodulin 1 up-regulated in both states; Cabin1 (calcineurin binding protein) up-regulated with high magnesium). cAMP response element binding proteins also showed a response to magnesium (CREB1 down-regulated by low magnesium; CREB3 up-regulated with high magnesium).

High magnesium conditions resulted in differential expression of 296 transcripts associated with cellular organisation and biogenesis (Table 3 and Table 4). Of these 54 were also differentially expressed with magnesium deficiency, with all but 3 responding in the same manner (up- or down-regulation) in both states. The exceptions were Tfb1m, Col4A2 and Arhgap18. In magnesium-depleted cells the changes were mostly related to cell component biogenesis (assembly of macromolecules and cell components), whilst magnesium supplementation had a larger impact on organelles and their organisation (including cytoskeletal effects).

### 3.3. Effects of Magnesium Concentration on Genes Involved in Inflammation, Mediated by the Chemokine and Cytokine Signalling Pathway

As previous studies had demonstrated that variation in magnesium concentration altered the response of HUVECs to inflammatory challenges we focused analysis on pathways involved in inflammation. Interleukin-8 (IL-8) was one of the transcripts shown to be responsive to low and high magnesium concentrations in culture. Given this and the fact that inflammation plays a key role in endothelial dysfunction, we examined the effects of magnesium on IL-8 associated inflammatory processes using the Gene Ontology (GO) Consortium bioinformatics resource. High-magnesium treatment significantly affected 444 transcripts that respond to stress, 33 of which were involved in inflammation mediated by the chemokine and cytokine signaling pathway (Table 5). Ten of these, including IL-8, were involved in the interleukin signaling pathway (Table 6). In contrast, the low-magnesium treatment significantly altered mRNA expression of 376 genes that respond to stress, 28 of which belonged to the inflammation mediated by chemokine and cytokine signaling pathway (Table 7). Five of these genes were a part of the interleukin signaling pathway (Table 8).

## 4. Discussion

Magnesium deficiency has been reported to be associated with a greater risk of cardiovascular disease in humans, and animal studies have shown that it promotes changes to arterial architecture and exacerbates atherosclerosis in transgenic strains [12,17,28]. Vascular endothelial cells play a central role in the development of atherosclerosis, and there has been interest in whether magnesium status is associated with cardiovascular risk due to the development of endothelial dysfunction in magnesium deficient states. We and others have previously shown that growing HUVECs under acute conditions of magnesium deficiency results in an exaggerated inflammatory response [16,29]. Our previous work reported greater expression of cytokines and adhesion molecules both before and after endotoxin challenge, and that high magnesium concentrations greatly blunted the inflammatory response [30]. The aim of the current work was to evaluate the extent of the HUVEC transcriptome response to variation in magnesium concentration. This provides a greater mechanistic understanding of the association between magnesium and endothelial cell dysfunction and identifies priorities for future study. The work is novel as few studies have evaluated the impact of magnesium on the whole transcriptome in human cells. Martin et al., reported that the impact of lifelong magnesium deficiency or supplementation had only modest effects on the rat liver transcriptome, but this study did not consider acute effects of magnesium, the response to further physiological challenges and looked at a whole tissue rather than specific cell type [31].

Findings presented in this paper show that 12.3% of the transcripts on the microarray were responsive to magnesium treatment, with 6.5% changing due to high magnesium treatment and the remaining 5.8% changing due to low magnesium treatment. In addition, 54% of the transcripts that were affected by high magnesium were up-regulated, and 46% were down-regulated, while 47% of the low magnesium group transcripts were up-regulated, and 52% were down-regulated. These data suggest that magnesium has a powerful effect on the HUVEC transcriptome. Importantly, the study found that magnesium can alter a high percentage of HUVECs genes involved in essential physiological pathways and highlighted the critical role of magnesium in inflammation. In comparison, Nicholson, Tucker and Brameld [5] found that around 6% of the genome was differentially expressed when HUVECs were treated with three polyphenolic compounds, ferulic acid, quercetin and resveratrol. In contrast, Chen, et al. [32] found that only 1.5% of the HUVECs genome was impacted by E coli derived LPS. Bal, et al. [33] stimulated HUVECs with different interleukins and found that stimulation of HUVECs with IL-1β changed 1% of the gene profile, while IL-3 affected 1.4% and IL-6 changed around 1.5% of the HUVECs genome. 

The analysis showed that varying the magnesium concentration in culture medium had effects across all cellular components, including the cytosol, nucleus and other organelles. Given the large numbers of transcripts responding in the experiment and the fundamental role of magnesium in processes such as binding to ATP in enzyme catalysed reactions, synthesis of nucleic acids and stabilizing membranes, this finding was perhaps unsurprising. The impact upon such basic processes will have fed into other aspects of the response to magnesium as categorised as ‘cellular processes’ and ‘cellular organisation’ by the GO analysis. 

Our previous work [30] and a robust body of literature [15,17,34] demonstrate that magnesium deficiency is detrimental to the function of HUVECs in culture, in particular reducing cell proliferation, increasing cell death, and exacerbating the response to inflammatory insults. The findings of the current study are consistent with such observations as they suggest perturbation of inflammatory pathways and apoptosis. The phenotype that we have previously shown to be associated with high magnesium under inflammatory conditions includes a dampened down inflammatory response, reduced cell death, and suppression of adhesion to monocytes [30]. It was apparent from the current experiment that modulation of intracellular signaling, apoptosis, binding processes, cell adhesion, and metabolic activities was widespread even in the absence of inflammatory challenge. These responses can all be envisaged as contributing to such a phenotype. The clear lack of dose-responses to magnesium was an interesting aspect of the study. Only 34 transcripts that responded to magnesium showed evidence of differential responses with magnesium deficiency and excess. These did not cluster in any process or pathway. With almost 20% of the transciptomic response to high magnesium being the same as with magnesium deficiency, it is clear that excess magnesium could be detrimental to HUVEC function.

The key starting point for this study was that magnesium regulates endothelial cell function and modulates the response to an inflammatory challenge [4,28,35]. Analysis of the array results demonstrated that mRNA expression for many of the interleukin genes, such as IL8, IL5, IL33, IL6R, IL1RL1, and IL31RA, and chemokine genes, such as CXCL6 (GCP-2), CXCL1 (GROα) and CXCL2 (GROβ), was down-regulated by high magnesium treatment, consistent with the hypothesis that maintaining magnesium concentrations suppresses the inflammatory response in HUVECs. In contrast, and at variance with our previous finding that expression of cytokine proteins such as IL-8, MCP-1, GRO, GROα, IL-2 and IL-3 was enhanced with magnesium deficiency [14], the array analysis found little impact of low magnesium on interleukins and their associated signaling pathway. This inconsistency may be because some effects of magnesium deficiency are mediated at the level of translation rather than transcription. The microarray data and associated GO analysis has clearly indicated that magnesium is a potent modulator of gene expression in HUVECs and that inflammatory processes are among those which are responsive to variation in magnesium concentration.

The heat map for the 615 transcripts that were commonly differentially expressed in both high and low magnesium treatment showed that the physiological magnesium concentration group matrix clustered differently between the high and low groups and that, surprisingly, the majority of these transcripts responded in the same manner in both conditions. Further analysis of these transcripts found that 13% of these genes were related to the cytoskeleton, and 17% and 52% were involved in anion and protein binding, respectively. Magnesium plays a role in cytoskeleton formation during the mitotic spindle and cytokinesis [36], and the present data suggest that deviation away from optimal concentration (either deficiency or excess) can disrupt this process. Moreover, any variation in the magnesium concentration can affect the other cell ions, such as Ca^2+^, Na^+,^ and K^+^ [36,37]. The efflux of Ca^2+^ can be influenced by the concentration of Mg^2+^ on the membrane binding sites because the magnesium ion is a Ca^2+^ antagonist. In addition, K^+^ transport is controlled by Mg^2+^ through the Na^+^-K^+^ ATPase pump. Further, extracellular magnesium can impact the ionized and protein bonds [38]. 

With 20% of all of the transcripts that responded to magnesium showing the same response in both low and high magnesium conditions, it appears that some of the effects of magnesium may be indirect and related to other factors. Magnesium shares key transporters with calcium, particularly TRPM6 and TRMP7 [39]. The latter is also a zinc transporter. The effects of low and high magnesium concentrations on the overall intracellular ion balance of the cell was not evaluated in this study, but would certainly be of interest. We noted that key components of the apparatus which enables calcium to regulate gene expression were perturbed by both low and high magnesium. Calcium binding to calmodulin (expressed in our study) and calcineurin activates the nuclear factor of activated T-cells family of transcription factors (NFATs-differentially regulated in the current study) leading to transcription. The ryanodine receptors (Ryr1 and Ryr2 responded to magnesium concentration in this study) regulate the release of calcium from intracellular stores enabling calmodulin to activate CREB sites on DNA.

It is important to acknowledge that this was an exploratory study that has been useful in generating hypotheses for further studies and demonstrating the complexity of the cellular response to variation in magnesium concentrations. We acknowledge that changes at the transcriptomic level do not necessarily reflect changes at the protein or functional level. We have shown elsewhere that the differential mRNA expression described in the inflammatory pathways reported in this paper are matched by differential expression (to a similar degree) in the protein expression of interleukins (IL-2, IL-3, IL-8, IL-15) and MCP-1 [14,30]. This confirms observations in the current work. The diverse range of pathways and processes that were responsive to magnesium was surprising, particularly given the acute challenge, although the magnitude of the expression changes was relatively small in most cases. This is not unusual in studies where nutrients are the stimulus or insults applied to cells and tissues. It is unclear whether the same differences in expression associated with low and/or high magnesium in culture would be present in the presence of an inflammatory insult and this requires further investigation either using a similar whole genome or a more targeted approach. Certainly the finding that IL-1β for example was down-regulated in conditions of low magnesium, differs from previous reports that low magnesium alone results in up-regulation [40,41]. Our data is consistent with the view that acute variation in magnesium concentration establishes the conditions for an altered response to inflammatory challenge [30,32] and we have previously reported that the combined insult may differ from the effects of varying magnesium concentration alone [30].

## 5. Conclusions

This study has shown that the expression of transcripts in HUVECs is extremely sensitive to the concentration of magnesium in culture. Considered in the context of observations that magnesium deficiency is associated with an exaggerated inflammatory response in this cell type, whilst high magnesium has a protective effect, it can be inferred that fluctuations in magnesium concentration are sensed within cells and modulate gene expression in diverse pathways. The data suggests that there are a number of responses to magnesium that may be indirectly mediated through variation in intracellular concentrations of other ions. However, magnesium should be recognised as an important factor in determining inflammatory responses in endothelial cells, and this may explain reports of associations between poor magnesium status and cardiovascular disease in humans and animals.

## Figures and Tables

**Table 1:** List of top 20 genes based on fold change that were differentially expressed in HUVECs culture in 0.1 mM MgSO_4_.

Prob Set Name	Entrez Gene	Symbol	Gene Title	*p*-Value	Log 2 Fold-Change
225160_x_at	4193	MDM2	Mdm2 p53 binding protein homolog (mouse)	0.046	2.1
231108_at	2521	FUS	Fused in sarcoma	0.031	1.7
232423_at	414	ARSD	Arylsulfatase D	0.038	1.7
215123_at	23,117	NPIPL3	Nuclear pore complex interacting protein-like 3	0.031	1.7
244872_at	5928	RBBP4	retinoblastoma binding protein 4	0.023	1.6
213742_at	9295	SRSF11	Serine/arginine-rich splicing factor 11	0.046	1.6
209936_at	10,181	RBM5	RNA binding motif protein 5	0.021	1.5
232898_at	1601	DAB2	Disabled homolog 2, mitogen-responsive phosphoprotein	0.003	1.5
243599_at	100,507,226	LOC100507226	Hypothetical LOC100507226	0.04	1.5
216983_s_at	7767	ZNF224	Zinc finger protein 224	0.008	1.4
224576_at	57,222	ERGIC1	Endoplasmic reticulum-golgi intermediate compartment (ERGIC) 1	0.045	−1.7
211611_s_at	1388///7148	ATF6B	Activating transcription factor 6 beta	0.045	−1.7
1555561_a_at	55,757	UGGT2	UDP-glucose glycoprotein glucosyltransferase 2	0.001	−1.6
210932_s_at	6049	RNF6	Ring finger protein (C3H2C3 type) 6	0.023	−1.5
229667_s_at	3218	HOXB8	Homeobox B8	0.024	−1.5
242157_at	80,205	CHD9	Chromodomain helicase DNA binding protein 9	0.019	−1.5
1555106_a_at	51,496	CTDSPL2	CTD (carboxy-terminal domain, RNA polymerase II, polypeptide A) small phosphatase like	0.042	−1.5
221440_s_at	10,741	RBBP9	Retinoblastoma binding protein 9	0.001	−1.4
213606_s_at	396	ARHGDIA	Rho GDP dissociation inhibitor (GDI) alpha	0.042	−1.4
206288_at	5229	PGGT1B	Protein geranylgeranyltransferase type I, beta subunit	0.001	−1.4

**Table 2:** List of top 20 genes based on fold change that were differentially expressed in HUVECs culture in 5 mM MgSO_4_.

Prob Set Name	Entrez Gene	Gene Symbol	Gene Title	*p*-Value	Log2 Fold-Change
228656_at	5629	PROX1	Prospero homeobox 1	0.001	1.6
205410_s_at	493	ATP2B4	ATPase, Ca++ transporting, plasma membrane 4	0.01	1.5
209401_s_at	6560	SLC12A4	Solute carrier family 12 (potassium/chloride transporters), member 4	0.034	1.5
241985_at	133,746	JMY	Junction mediating and regulatory protein, p53 cofactor	0.04	1.4
243323_s_at	463	ZFHX3	Zinc finger homeobox 3	0.002	1.4
221085_at	9966	TNFSF15	Tumor necrosis factor (ligand) superfamily, member 15	0.018	1.4
207332_s_at	7037	TFRC	Transferrin receptor (p90, CD71)	*p* < 0.001	1.4
219772_s_at	23,676	SMPX	Small muscle protein, X-linked	0.004	1.4
216950_s_at	100,132,417///2209	FCGR1A	Fc fragment of IgG, high affinity Ia, receptor (CD64)	0.018	1.4
210954_s_at	9819	TSC22D2	TSC22 domain family, member 2	0.038	1.3
236361_at	117,248	GALNTL2	UDP-N-acetyl-alpha-D-galactosamine	*p* < 0.001	−2.0
1556325_at	27,145	FILIP1	Filamin A interacting protein 1	*p* < 0.001	−1.9
206336_at	6372	CXCL6	Chemokine (C-X-C motif) ligand 6	*p* < 0.001	−1.8
230543_at	8239	USP9X	Ubiquitin specific peptidase 9, X-linked	0.039	−1.7
240757_at	23,332	CLASP1	Cytoplasmic linker associated protein 1	0.007	−1.6
203889_at	6447	SCG5	Secretogranin V	0.004	−1.6
1570515_a_at	27,145	FILIP1	Filamin A interacting protein 1	*p* < 0.001	−1.6
219825_at	56,603	CYP26B1	Cytochrome P450, family 26, subfamily B, polypeptide 1	0.004	−1.5
207542_s_at	358	AQP1	Aquaporin 1 (Colton blood group)	*p* < 0.001	−1.5
211506_s_at	3576	IL8	Interleukin 8	0.011	−1.5

**Table 3:** GO enrichment analysis of transcripts regulated by high magnesium concentration. Analysis applied FDR *p* < 0.005 for enrichment *p* values.

GO Processes	Enrichment Score	Enrichment *p* Value	Number of Transcripts Differentially Regulated
**Biological processes**			
Cellular process	1.19	3.71 × 10^−9^	1052
Sensory perception of chemical signals	0.20	2.21 × 10^−5^	6
Metabolic process	1.19	2.64 × 10^−5^	748
Cellular component organisation or biogenesis	1.31	2.87 × 10^−4^	296
Primary metabolic process	1.18	4.31 × 10^−4^	604
Cell adhesion	1.78	4.94 × 10^−4^	68
Phosphate containing compound metabolic process	1.33	9.64 × 10^−4^	228
Intracellular signal transduction	1.39	2.22 × 10^−3^	160
Cell death	1.70	2.90 × 10^−3^	65
Apoptotic process	1.72	3.46 × 10^−3^	62
G-protein coupled receptor signalling pathway	0.49	3.46 × 10^−3^	24
Nucleobase-containing compound metabolic processes	1.21	3.69 × 10^−3^	364
**Molecular functions**			
Catalytic activity	1.21	4.30 × 10^−4^	547
Protein binding	1.23	6.15 × 10^−3^	365
Binding	1.15	8.45 × 10^−3^	608
Hydrolase activity	1.27	3.14 × 10^−4^	251
**Cellular components**			
Intracellular	1.23	1.91 × 10^−7^	694
Cell part	1.21	4.49 × 10^−7^	720
Cytoplasm	1.25	4.99 × 10^−5^	427
Organelle	1.21	1.71 × 10^−4^	507
Nucleus	1.27	1.91 × 10^−3^	265

**Table 4:** GO enrichment analysis of transcripts regulated by low magnesium concentration. The analysis applied FDR *p* < 0.005 for enrichment *p* values.

GO Processes	Enrichment Score	Enrichment *p* Value	Number of Transcripts Differentially Regulated
**Biological processes**			
Metabolic process	1.15	4.75 × 10^−3^	590
Cellular component organisation or biogenesis	1.33	1.02 × 10^−3^	243
Cellular process	1.14	3.73 × 10^−4^	819
**Cellular components**			
Ribosome macromolecular complex	1.32	3.24 x10^−4^	247
Cytoplasm	1.29	3.08 × 10^−5^	355
Intracellular	1.27	2.39 × 10^−8^	583
Nucleus	1.32	6.89 × 10^−4^	223
Organelle	1.24	3.94 × 10^−5^	424

**Table 5:** Differential expression of 33 transcripts based on fold change annotated to genes that are involved in inflammation mediated by chemokine and cytokine signaling pathway (High magnesium condition).

Prob Set Name	Entrez Gene	Gene Symbol	Gene Title	Gene Ontology Biological Process	*p*-Value	Log2 Fold-Change
207445_s_at	10,803	CCR9	Chemokine (C-C motif) receptor 9	Chemotaxis/inferred from electronic annotation/chemotaxis	0.042	1.3
211230_s_at	5293	PIK3CD	Phosphoinositide-3-kinase, catalytic, delta polypeptide	B cell homeostasis/inferred from electronic annotation/	0.004	1.3
216834_at	5996	RGS1	Regulator of G-protein signaling 1	Immune response/traceable author statement/signal transduce	0.047	1.2
230202_at	5970	RELA	Transcription factor p65	Liver development/inferred from electronic annotation	0.047	1.2
221244_s_at	5170	PDPK1	3-phosphoinositide dependent protein kinase-1	Protein phosphorylation/inferred from electronic annotation/	0.022	1.2
224909_s_at	57,580	PREX1	Phosphatidylinositol-3,4,5-trisphosphate-dependent Rac exchange factor 1	Superoxide metabolic process/traceable author statement	0.019	1.1
210162_s_at	4772	NFATC1	Nuclear factor of activated T-cells, cytoplasmic, calcineurin-dependent 1	G1/S transition of mitotic cell cycle/inferred from electronic annotation	0.036	1.1
222912_at	408	ARRB1	Arrestin, beta 1	G-protein coupled receptor internalization/inferred from mutant phenotype	0.015	1.1
203175_at	391	RHOG	Rho-related GTP-binding protein, member G (rho G)	Small GTPase mediated signal transduction/inferred from electronic annota	0.035	1.1
212777_at	6654	SOS1	Son of sevenless homolog 1	Apoptosis/not recorded signal transduction	0.017	1.1
211543_s_at	2870	GRK6	G protein-coupled receptor kinase 6	Protein phosphorylation/inferred from electronic annotation	0.006	1.1
205884_at	3676	ITGA4	Integrin, alpha 4 (antigen CD49D, alpha 4 subunit of VLA-4 receptor)	Blood vessel remodeling inferred from electronic annotation	0.029	1.1
205127_at	5742	PTGS1	Prostaglandin-endoperoxide synthase 1	Prostaglandin biosynthetic process/inferred from sequence or structural s	0.017	1.1
33814_at	10,298	PAK4	P21 protein (Cdc42/Rac)-activated kinase 4	Protein phosphorylation/inferred from electronic annotation	0.019	1.1
228388_at	4793	NFKBIB	Nuclear factor of kappa light polypeptide gene enhancer in B-cells inhibitor, beta	Transcription/traceable author statement/signal transducti	0.01	1.1
200885_at	389	RHOC	Rho-related GTP binding protein, member C	Small GTPase mediated signal transduction/inferred from electronic annota	0.018	1.1
208075_s_at	6354	CCL7	Chemokine (C-C motif) ligand 7	Cellular calcium ion homeostasis/traceable author statement	0.042	1.1
201188_s_at	3710	ITPR3	Inositol 1,4,5-triphosphate receptor, type 3	Transport/inferred from electronic annotation/ion transport	0.038	1.1
225363_at	5728	PTEN	Phosphatase and tensin homolog	Regulation of cyclin-dependent protein kinase activity/traceable authors	0.03	1.1
205125_at	5333	PLCD1	Phospholipase C, delta 1	Lipid metabolic process/inferred from electronic annotation/	0.03	1.1
201895_at	369	ARAF	Serine/threonine protein kinase A raf	Protein modification process/traceable author statement	*p* < 0.001	1.1
212647_at	6237	RRAS	Related RAS viral (r-ras) oncogene homolog	Signal transduction/inferred from electronic annotation	0.041	1.1
224994_at	817	CAMK2D	Calcium/calmodulin-dependent protein kinase II delta	G1/S transition of mitotic cell cycle//inferred from electronic annotation	0.038	1.1
206336_at	6372	CXCL6	Chemokine (C-X-C motif) ligand 6 (granulocyte chemotactic protein 2)	Chemotaxis/inferred from electronic annotation/chemotaxis	*p* < 0.001	−1.8
211506_s_at	3576	IL8	Interleukin 8	Angiogenesis/traceable author statement/cellular component	0.011	−1.5
204470_at	2919	CXCL1	Chemokine (C-X-C motif) ligand 1 (melanoma growth stimulating activity, alpha)	Chemotaxis/traceable author statement inflammatory response	0.027	−1.2
225139_at	4775	NFATC3	Nuclear factor of activated T-cells, cytoplasmic, calcineurin-dependent 3	Transcription/inferred from electronic annotation	0.033	−1.0
204010_s_at	3845	KRAS	GTPase KRas	Apoptosis/inferred from electronic annotation/signal trans	0.029	−1.1
38290_at	10,636	RGS14	Regulator of G-protein signaling 14	Mitosis/inferred from electronic annotation/signal transduce	0.001	−1.1
204103_at	6351	CCL4	Chemokine (C-C motif) ligand 4	Cellular component movement/traceable author statement	0.008	−1.1
207952_at	3567	IL5	Interleukin 5 (colony-stimulating factor, eosinophil)	Inflammatory response/inferred from electronic annotation	0.024	−1.1
213044_at	6093	ROCK1	Rho-associated, coiled-coil containing protein kinase 1	Cytokinesis/inferred from electronic annotation/protein ph	0.002	−1.1
209774_x_at	2920	CXCL2	Chemokine (C-X-C motif) ligand 2	Chemotaxis/inferred from electronic annotation/chemotaxis	0.045	−1.1

**Table 6:** Differential expression of 10 transcripts based on fold change annotated to genes that are involved the interleukin signalling pathway (High magnesium condition).

Prob Set Name	Entrez Gene	Gene Symbol	Gene Title	Gene Ontology Biological Process	*p*-Value	Log2 Fold-Change
221244_s_at	5170	PDPK1	3-phosphoinositide dependent protein kinase-1	Protein phosphorylation/inferred from electronic annotation	0.022	1.2
228388_at	4793	NF-κB IB	Nuclear factor of kappa light polypeptide gene enhancer in B-cells inhibitor, beta	Transcription/traceable author statement/signal transducti	0.01	1.1
202284_s_at	1026	CDKN1A	Cyclin-dependent kinase inhibitor 1A (p21, Cip1)	Regulation of cyclin-dependent protein kinase activity/traceable authors	0.006	1.1
205170_at	6773	STAT2	Signal transducer and activator of transcription 2	Transcription/inferred from electronic annotation	0.04	1.0
211506_s_at	3576	IL8	Interleukin 8	Angiogenesis/traceable author statement//cellular component	0.011	−1.5
234066_at	9173	IL1RL1	Interleukin 1 receptor-like 1	Immune response/non-traceable author statement/signal tran	*p* < 0.001	−1.3
243541_at	133,396	IL31RA	Interleukin 31 receptor A	MAPKKK cascade/non-traceable author statement anti-apoptos	0.028	−1.2
207952_at	3567	IL5	Interleukin 5 (colony-stimulating factor, eosinophil)	Inflammatory response/inferred from electronic annotation	0.024	−1.2
209821_at	90,865	IL33	Interleukin 33	Positive regulation of macrophage activation/inferred from direct assay	0.014	−1.2
226333_at	3570	IL6R	Interleukin 6 receptor	Hepatic immune response/traceable author statement	0.044	−1.1

**Table 7:** Differential expression of 28 transcripts based on fold change annotated to genes that are involved in inflammation mediated by chemokine and cytokine signalling pathway (Low magnesium condition).

Prob Set Name	Entrez Gene	Gene Symbol	Gene Title	Gene Ontology Biological Process	*p*-Value	Log2 Fold-Change
211230_s_at	5293	PIK3CD	Phosphoinositide-3-kinase, catalytic, delta polypeptide	B cell homeostasis/inferred from electronic annotation	0.006	1.3
205127_at	5742	PTGS1	Prostaglandin-endoperoxide synthase 1 (prostaglandin G/H synthase and cyclooxygenase)	Prostaglandin biosynthetic process/inferred from sequence or structural	0.008	1.2
221244_s_at	5170	PDPK1	3-phosphoinositide dependent protein kinase-1	Protein phosphorylation/inferred from electronic annotation	0.01	1.2
201188_s_at	3710	ITPR3	Inositol 1,4,5-triphosphate receptor, type 3	Transport/inferred from electronic annotation ion transpor	0.03	1.2
233254_x_at	5728	PTEN	Phosphatase and tensin homolog	Regulation of cyclin-dependent protein kinase activity/traceable authors	0.006	1.2
232043_at	2788	GNG7	Guanine nucleotide binding protein (G protein), gamma 7	Behavioral fear response/inferred from electronic annotation	0.03	1.1
1568926_x_at	91,807	MYLK3	Myosin light chain kinase 3	Protein phosphorylation/inferred from electronic annotation	0.018	1.1
216190_x_at	3688	ITGB1	Integrin, beta 1 (fibronectin receptor, beta polypeptide, antigen CD29 includes MDF2, M	G1/S transition of mitotic cell cycle/inferred from electronic annotation	0.014	1.1
224994_at	817	CAMK2D	Calcium/calmodulin-dependent protein kinase II delta	G1/S transition of mitotic cell cycle//inferred from electronic annotation	0.01	1.1
205962_at	5062	PAK2	P21 protein (Cdc42/Rac)-activated kinase 2	Protein phosphorylation/inferred from direct assay/protein	0.017	1.1
243829_at	673	BRAF	V-raf murine sarcoma viral oncogene homolog B1	MAPKKK cascade/inferred from electronic annotation/protein	0.003	1.1
210162_s_at	4772	NFATC1	Nuclear factor of activated T-cells, cytoplasmic, calcineurin-dependent 1	G1/S transition of mitotic cell cycle/inferred from electronic annotation	0.04	1.1
209201_x_at	7852	CXCR4	Chemokine (C-X-C motif) receptor 4	Activation of MAPK activity traceable author statement	0.01	−1.3
1560524_at	400,581	GRAP	GRB2-related adaptor protein	Ras protein signal transduction/traceable author statement	0.03	−1.2
224965_at	54,331	GNG2	Guanine nucleotide binding protein (G protein), gamma 2	Signal transduction/inferred from electronic annotation	0.01	−1.2
208641_s_at	5879	RAC1	Ras-related C3 botulinum toxin substrate 1 (rho family, small GTP binding protein Rac1)	Endocytosis/inferred from electronic annotation/apoptosis	0.01	−1.1
204174_at	241	ALOX5AP	Arachidonate 5-lipoxygenase-activating protein	Leukotriene production involved in inflammatory response/inferred from el	0.04	−1.1
222912_at	408	ARRB1	Arrestin, beta 1	G-protein coupled receptor internalization/inferred from mutant phenotype	0.04	−1.1
201179_s_at	2773	GNAI3	Guanine nucleotide binding protein (G protein), alpha inhibiting activity polypeptide 3	Transport/non-traceable author statement/vesicle fusion/	0.007	−1.1
212590_at	22,800	RRAS2	Related RAS viral (r-ras) oncogene homolog 2	Signal transduction/inferred from electronic annotation	0.013432	−1.1
204103_at	6351	CCL4	Chemokine (C-C motif) ligand 4	Cellular component movement/traceable author statement	0.01	−1.1
225141_at	4775	NFATC3	Nuclear factor of activated T-cells, cytoplasmic, calcineurin-dependent 3	Transcription/inferred from electronic annotation	0.036	−1.1
202647_s_at	4893	NRAS	Neuroblastoma RAS viral (v-ras) oncogene homolog	Apoptosis/inferred from electronic annotation/signal trans	0.005	−1.1
211434_s_at	9034	CCRL2	Chemokine (C-C motif) receptor-like 2	Chemotaxis/traceable author statement/signal transduction	0.03	−1.1
39402_at	3553	IL1B	Interleukin 1, beta	Activation of MAPK activity/inferred from direct assay	0.02	−1.1
203154_s_at	10,298	PAK4	P21 protein (Cdc42/Rac)-activated kinase 4	Protein phosphorylation/inferred from electronic annotation	0.03	−1.05
38290_at	10,636	RGS14	Regulator of G-protein signaling 14	Mitosis/inferred from electronic annotation/signal transdu	0.004	−1.0
207157_s_at	2787	GNG5	Guanine nucleotide binding protein (G protein), gamma 5	Signal transduction/non-traceable author statement/signal	0.03	−1.0

**Table 8:** Differential expression based on fold change of 5 transcripts annotated to genes that are involved the interleukin signalling pathway (Low magnesium condition).

Prob Set Name	Entrez Gene	Gene Symbol	Gene Title	Gene Ontology Biological Process	*p*-Value	Log2 Fold-Change
204906_at	6196	RPS6KA2	Ribosomal protein S6 kinase, 90 kDa, polypeptide 2	Mitotic metaphase/inferred from electronic annotation	0.01	1.2
221244_s_at	5170	PDPK1	3-phosphoinositide dependent protein kinase-1	Protein phosphorylation/inferred from electronic annotation	0.01	1.2
243829_at	673	BRAF	V-raf murine sarcoma viral oncogene homolog B1	MAPKKK cascade/inferred from electronic annotation/protein	0.003	1.1
202647_s_at	4893	NRAS	Neuroblastoma RAS viral (v-ras) oncogene homolog	Apoptosis/inferred from electronic annotation/signal trans	0.005	−1.1
207538_at	3565	IL4	Interleukin 4	Chemotaxis/traceable author statement/immune response	0.04	−1.1

## Data Availability

The datasets used and analyzed during the current study are available from the corresponding author upon reasonable request.

## Supplementary Materials

The following supporting information can be downloaded at: https://www.mdpi.com/article/10.3390/nu14173586/s1, Supplementary Table S1: Transcripts showing differential expression in response to high magnesium concentration; Supplementary Table S2: 615 transcripts showing differential expression to both low- and high-magnesium concentrations.
